# The “Goldilocks Zone” from a redox perspective—Adaptive vs. deleterious responses to oxidative stress in striated muscle

**DOI:** 10.3389/fphys.2014.00358

**Published:** 2014-09-18

**Authors:** Rick J. Alleman, Lalage A. Katunga, Margaret A. M. Nelson, David A. Brown, Ethan J. Anderson

**Affiliations:** ^1^Departments of Physiology, East Carolina UniversityGreenville, NC, USA; ^2^East Carolina Diabetes and Obesity Institute, East Carolina UniversityGreenville, NC, USA; ^3^Pharmacology and Toxicology, Brody School of Medicine, East Carolina UniversityGreenville, NC, USA

**Keywords:** hormesis, carbonyl stress, mitochondria, lipid peroxidation, redox environment, adaptation, heart, skeletal muscle

## Abstract

Consequences of oxidative stress may be beneficial or detrimental in physiological systems. An organ system's position on the “hormetic curve” is governed by the source and temporality of reactive oxygen species (ROS) production, proximity of ROS to moieties most susceptible to damage, and the capacity of the endogenous cellular ROS scavenging mechanisms. Most importantly, the resilience of the tissue (the capacity to recover from damage) is a decisive factor, and this is reflected in the disparate response to ROS in cardiac and skeletal muscle. In myocytes, a high oxidative capacity invariably results in a significant ROS burden which in homeostasis, is rapidly neutralized by the robust antioxidant network. The up-regulation of key pathways in the antioxidant network is a central component of the hormetic response to ROS. Despite such adaptations, persistent oxidative stress over an extended time-frame (e.g., months to years) inevitably leads to cumulative damages, maladaptation and ultimately the pathogenesis of chronic diseases. Indeed, persistent oxidative stress in heart and skeletal muscle has been repeatedly demonstrated to have causal roles in the etiology of heart disease and insulin resistance, respectively. Deciphering the mechanisms that underlie the divergence between adaptive and maladaptive responses to oxidative stress remains an active area of research for basic scientists and clinicians alike, as this would undoubtedly lead to novel therapeutic approaches. Here, we provide an overview of major types of ROS in striated muscle and the divergent adaptations that occur in response to them. Emphasis is placed on highlighting newly uncovered areas of research on this topic, with particular focus on the mitochondria, and the diverging roles that ROS play in muscle health (e.g., exercise or preconditioning) and disease (e.g., cardiomyopathy, ischemia, metabolic syndrome).

## Introduction

**Figure 1:**
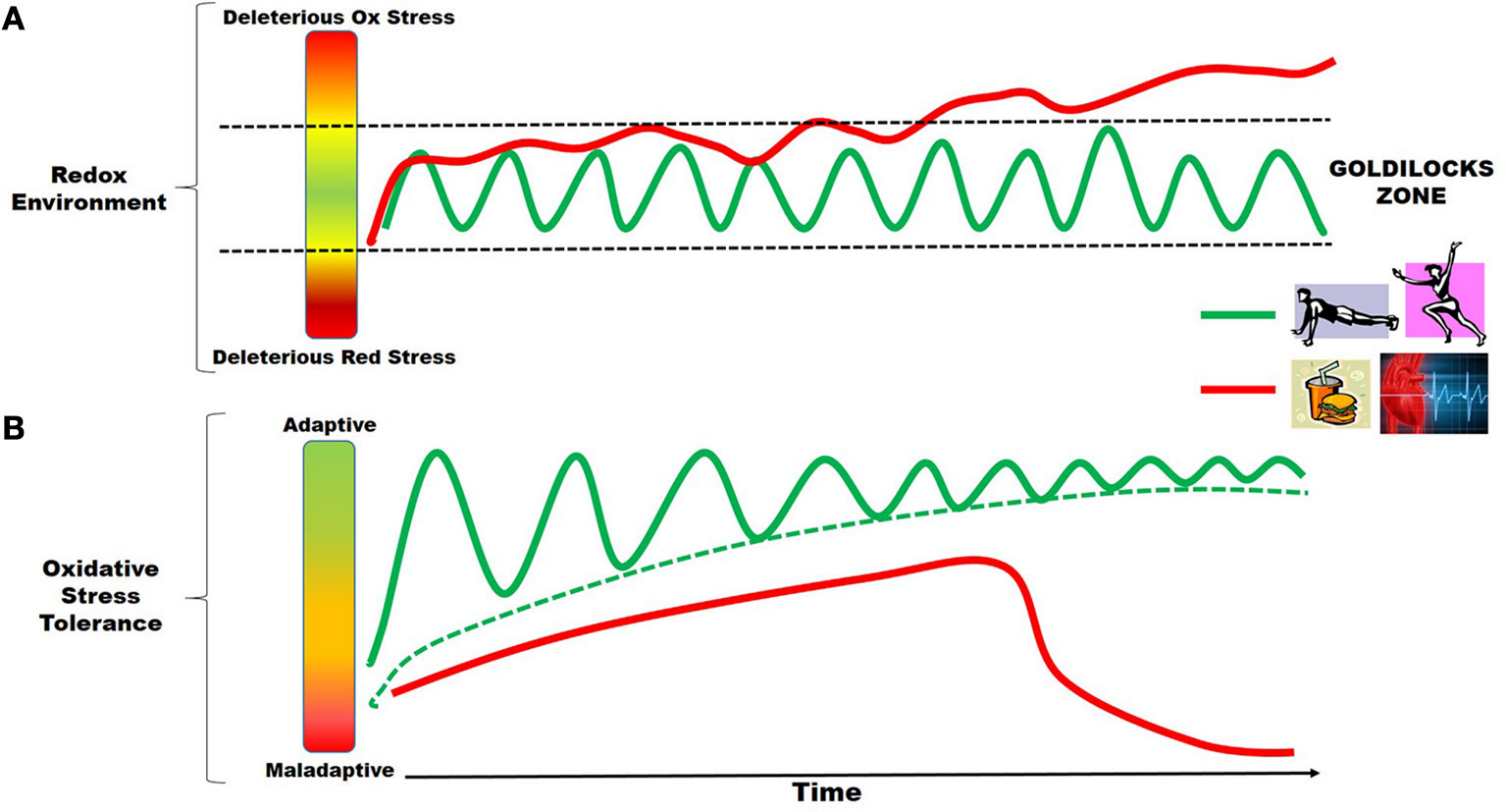
**Oxidative stress adaptations in striated muscle and the Goldilocks Zone**. The concept of the Goldilocks Zone in the redox environment of striated muscle is illustrated in the schematics shown above. Time-dependent changes in tissue oxidative stress due to exercise (green line) is pulsatile in nature, coming from consecutive bouts of exercise over time, whereas the oxidative stress arising from cardio-metabolic diseases (red line) is persistent. As depicted in **(A)**, the ultimate outcome of these two stressors is divergent, since exercise-induced ROS over time keeps the myocytes well within the Goldilocks Zone of homeostasis (region between the Reducing and Oxidative stress, two dashed black lines), while disease-induced ROS eventually pushes the myocytes outside of the Zone, leading to deleterious consequences (e.g., unresolved inflammation, electro-mechanical dysfunction, mitochondrial dysfunction and cell death). These divergent outcomes are best explained by the adaptive responses in the antioxidant capacity and cell quality control mechanisms that are elicited by these two sources of oxidative stress. **(B)** Illustrates this adaptive response, in that exercise-induced ROS leads to augmentation of the antioxidant capacity and protection against exogenous stressors over time. Conversely, disease-induced ROS, due to its persistent and insidious nature, ultimately overwhelms endogenous protective mechanisms, ultimately resulting in a maladaptive response.

A fairy tale emerged in England during the first half of the nineteenth century, telling the story of how a meddlesome little girl named Goldilocks discovered the forest home of a family of bears. In this classic tale, the reader will remember that during her exploration of the bears' home, Goldilocks determined that baby bear's porridge was “not too hot, not too cold,” and his bed was “not too hard, not too soft.” In essence, they were “just right.” Such is the nature of homeostasis in our organ systems, in that stress in either one direction or another can tip the scales toward disease, or maladaptation, thereby disrupting homeostasis. It is in this context that the redox environment of a cell is maintained within a “Goldilocks Zone,” whereby ROS production is sufficiently counterbalanced by the antioxidant capacity/quality control systems, and the environment is optimal for homeostasis. However, when faced with persistent or uncompensated oxidative stress, the redox environment can be pushed outside of this Goldilocks Zone, where cell death, inflammation and disease ensues. The schematic shown in Figure 1A illustrates this concept of the Goldilocks Zone in redox balance, and uses two contrasting stressors that are common to the cardiovascular system, cardio-metabolic disease and exercise, as examples. Both exercise and disease generate oxidative stress in striated muscle. However, the persistent and uncompensated oxidative stress resulting from cardio-metabolic disease ultimately pushes the myocytes outside the Goldilocks Zone, while the pulsatile, compensated oxidative stress that follows successive bouts of exercise keeps the myocytes in the Zone, and in good health.

High metabolic demand in striated muscle necessitates a far greater degree of arterial blood perfusion and oxygen tension than virtually any other tissue in the body. The natural consequence of this high availability and concentration of oxygen is a greater burden placed upon endogenous antioxidant systems to buffer the ROS that are invariably produced as a bi-product. Numerous enzymatic and non-enzymatic sources of ROS exist in skeletal muscle and heart, and it is only through the work of these important antioxidant networks that the cellular redox environment is maintained within a range that is optimal for homeostasis and proper electro-mechanical function. ROS production in striated muscle is persistent in nature, therefore, oxidative stress in this tissue should be viewed in the context of a continuum in that there is always a degree of oxidative stress present, and it only becomes deleterious under certain contexts (i.e., persistence, compartmentalization or lack of adaptive responses). In fact, recent work in transgenic mice has documented that the removal of NADPH oxidase (NOX) in the heart, and the subsequent presence of overwhelming reductive stress, can lead to exacerbation of ischemia/reperfusion injury (Matsushima et al., 2013). A similar phenomenon is observed in skeletal muscle, where overexpression of the antioxidant enzyme glutathione peroxidase-1 (GPx1) in mice was shown to cause insulin resistance (McClung et al., 2004). These paradoxical findings embody the “not too hot, not too cold” concept of the Goldilocks Zone in redox balance, and also underscore both the complexity and importance of the redox environment to homeostasis. Further, they are evidence of the need for a more rigorous investigation of the mechanisms by which the redox environment of striated muscle adapts to various metabolic challenges.

This paradox between “good” and “bad” oxidative stress is best exemplified in the contrasting effects of the increased ROS that is well-known to occur in striated muscle with exercise, compared to the increased ROS from cardio-metabolic diseases characterized by metabolic supply/demand mismatch, such as nutrient overload (i.e., obesity, high fat/high sugar diets, diabetes) and ischemia. Shown in Figure 1B is a schematic depicting how disease-induced ROS and exercise-induced ROS lead to divergent outcomes in striated muscle. Both cardio-metabolic disease and exercise cause increases in ROS, but they differ in their substance and temporality. The ROS produced from successive bouts of exercise is pulsatile in nature, with periods of recovery in between (green line Figure 1B). Indeed, increased ROS occurs in skeletal muscle and heart during exercise from a variety of sources, but the broad outcome of this oxidative stress is beneficial, resulting in the induction of mitochondrial biogenesis and aerobic capacity, augmented antioxidant capacity, improved vascularization, and insulin sensitivity. The beneficial ROS-mediated adaptations to exercise have been the topic of many thorough reviews (Powers and Jackson, 2008; Powers et al., 2010, 2011a,b; Gomez-Cabrera et al., 2012). In many ways the response of striated muscle to exercise-induced oxidative stress is embodied in the concept of hormesis, in that the muscle experiences a beneficial, adaptive response to sub-lethal increases in ROS that occur during and immediately following each bout of exercise (Ristow and Schmeisser, 2011; Nikolaidis et al., 2012). Conversely, nutrient overload and ischemia/reperfusion also cause increased ROS in skeletal muscle and heart from numerous sources, particularly mitochondria, but net effects are deleterious, resulting in inflammation and recruitment of immune cells, activation of pro-fibrotic gene expression and cardiac hypertrophy, increased cell death, and insulin resistance (reviewed in Fisher-Wellman and Neufer, 2012; Cavalera et al., 2014; Dai et al., 2014). Some evidence of modest hormesis has been reported in myocardium with nutrient overload/diabetes (Joyeux-Faure et al., 2006; Tocchetti et al., 2012; Fisher-Wellman et al., 2013; Rindler et al., 2013; Chen et al., 2014; Lejay et al., 2014), but ultimately these modest adaptive responses become overwhelmed, and the tissue/organ succumbs to the deleterious effects over time (red line in Figure 1B).

The “Goldilocks Zone” of redox balance in muscle scales down from whole tissue to mitochondrial level. Work by Aon and colleagues showed that rates of ROS emission (ROS produced—ROS scavenged) from mitochondria are substantially elevated when the mitochondrial redox environment is either highly reduced (e.g., nutrient overload, diabetes), or highly oxidized (e.g., reperfusion) (Aon et al., 2010; Cortassa et al., 2014). This increased ROS emission observed at both ends of the redox spectrum would presumably lead to different consequences at cell and tissue level, as the antioxidant capacity is considerably more compromised during a highly oxidized state than it is during a highly reduced state. At the level of cells and tissues, however, there are also many other considerations such as the temporality of the change in redox environment (i.e., acute vs. persistent) and the existing mechanisms of adaptation intrinsic to the cells experiencing the stress at a given time, all of which would dictate the global response to increased mitochondrial ROS emission.

In the following sections we provide an overview of this contrast in outcomes between the “bad” oxidative stress that occurs in skeletal muscle and heart as a result of diseases associated with metabolic supply/demand mismatch (e.g., nutrient overload/obesity, ischemia), and “good” oxidative stress that occurs in these organs with therapeutic interventions such as pre-conditioning and exercise. Major sources of ROS and their byproducts in these conditions will be discussed, along with potential mechanisms underlying the adaptive, beneficial responses and the maladaptive, deleterious responses. Emphasis will be placed on mitochondrial sources of ROS and mitochondrial antioxidant networks, as this organelle is recognized as the primary source, sink, and target of intracellular ROS in muscle. Furthermore, descriptions of lesser-known types of oxidative stress (e.g., lipid peroxidation and carbonyl stress) and relevant detoxification systems are another area of special emphasis, as these are increasingly recognized to be major players in ROS-mediated adaptation. Where appropriate, clinical and translational studies will be described.

## Cardio-metabolic disease and oxidative stress in striated muscle

**Figure 2:**
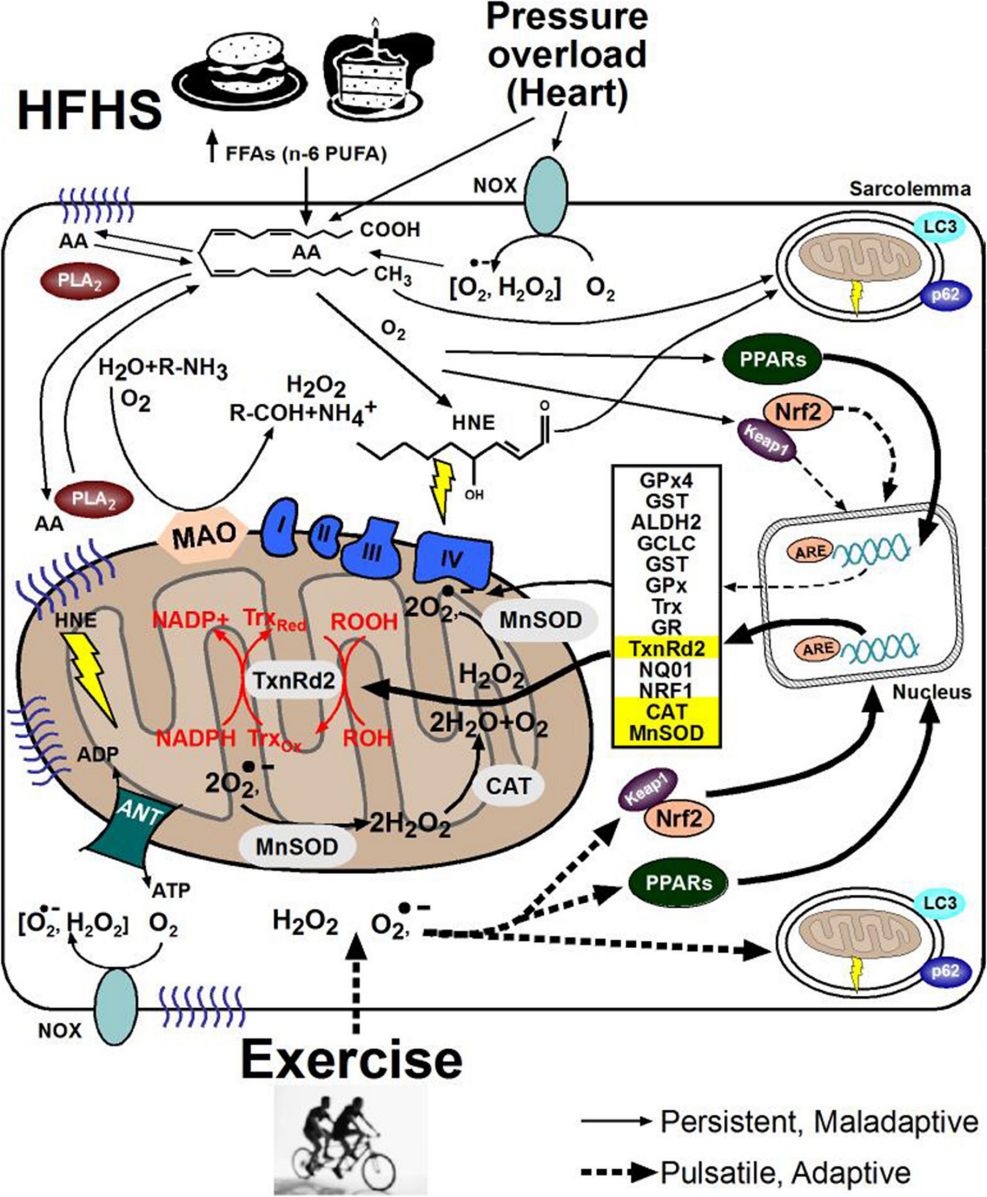
**Cell signaling pathways underlying ROS adaptation in striated muscle**. The beneficial and deleterious consequences of oxidative stress coming from cardio-metabolic diseases (top) or exercise (bottom) in striated muscle at the subcellular level are shown here. Note the difference in designation of solid-line arrows for the persistent ROS coming from disease, compared to dashed-line arrows representing the pulsatile ROS coming from exercise. Formation of lipid peroxides and their derivative aldehydes such as 4-hydroxynonenal (HNE), along with their subsequent reactivity to cause protein carbonylation and carbonyl stress is depicted by yellow lightning bolts in cytosol and mitochondria. (NOX, NADPH oxidase; HFHS, high fat, high sucrose; PUFA, polyunsaturated fatty acids; FFA, free fatty acids; AA, arachidonic acid; PLA_2_, phospholipase A_2_; PPAR, peroxisome proliferator-activated receptor; ANT, adenine nucleotide translocase; COX IV, cytochrome oxidase IV; ATP, adenosine tri-phosphate; Nrf2, NF E2-related factor 2; NRF1, nuclear respiratory factor 1; GST, glutathione S-transferase; ALDH2, aldehyde dehydrogenase 2; GCLC, γ-Glutamylcysteine ligase catalytic subunit; GPx, glutathione peroxidase; NQO-1, NADPH-quinone oxido-reductase-1; GR, glutathione reductase; GSHt, total glutathione; Trx, thioredoxin; TxnRd2, thioredoxin reductase-2; MAO, monoamine oxidase; MnSOD, manganese superoxide dismutase; CAT, catalase).

Much focus has been directed in recent years toward documenting the contribution of ROS “production” to disease etiology, and toward identifying enzymatic sources of ROS as underlying mechanisms. Such an emphasis toward the “supply-side” of ROS is important, but can often be short-sighted as it ignores the vast redox circuits and antioxidant networks that exist within cells. Indeed, a major source of the problem of oxidative stress in chronic disease likely involves the inability of the myocytes' antioxidant networks to adequately compensate for the increase in ROS production that occurs. Never-the-less, for purposes of this portion of the review we will focus on major sources of ROS production within myocytes, since a number of sources have consistently been shown to be associated with cardio-metabolic diseases such as obesity, diabetes, myocardial ischemia, and other chronic diseases. Whether this increased ROS is cause or consequence of these diseases is still not clear, although it appears likely that it is both. Figure 2 illustrates some of the major sources of ROS in myocytes which will be discussed in the subsequent sections, as well as common deleterious responses and adaptive responses that accompany the ROS. Understanding the mechanisms behind this lack of adaptability, or maladaptive response to ROS, may be the key to unraveling this paradox of “good vs. bad” oxidative stress in striated muscle, thereby leading to improvements in existing therapies and identification of novel therapeutic targets.

### Major sources of ROS production in striated muscle with cardio-metabolic diseases

#### Prominent mitochondrial and non-mitochondrial enzymatic sources of ROS

The cytosol, plasma membrane, nucleus, and peroxisomes are all sites of ROS production, but for myocytes in particular, the mitochondria is widely considered to be the predominant source of ROS (reviewed in Jezek and Hlavata, 2005). Within the mitochondria, the electron transport system (ETS) has been well-studied as a source of ROS production with superoxide radicals forming spontaneously at complexes I, II, and III via the swift addition of leaking electrons to an incompletely reduced oxygen (St-Pierre et al., 2002; Kudin et al., 2004; Drose and Brandt, 2012; Siebels and Drose, 2013). A comprehensive overview of the sites and regulation of ROS production by the mitochondrial ETS is beyond the scope of this review, and has been extensively reviewed elsewhere (Liu, 1997; Andreyev et al., 2005; Stowe and Camara, 2009).

Although the ETS is unquestionably a major producer of ROS in myocytes, the enzyme monoamine oxidase (MAO) is often overlooked as a mitochondrial source of ROS in cardiomyocytes. Tethered to the outer mitochondrial membrane, MAO is responsible for oxidative deamination of catecholamines, simultaneously generating H_2_O_2_, NH^+^_4_ and reactive aldehydes in the process (Lewinsohn et al., 1978). The two isoforms of the enzyme, MAO-A and MAO-B, have differing affinities for various substrates and inhibitors, although both isoforms can metabolize all catecholamines to some extent. MAO-A is selectively inhibited by clorgyline and has been shown to have a significantly greater capacity to metabolize serotonin and norepinephrine than MAO-B. MAO-B is selectively inhibited by selegiline and has a profoundly greater capacity to metabolize phenylethylamine then MAO-A. Both isoforms contribute relatively equally to oxidative deamination of dopamine, tyramine, and tryptamine (Youdim et al., 1988). The predominance of one isoform vs. the other within the body is tissue-dependent and the levels between species can also vary significantly (Saura et al., 1996). The heart contains a mix of both isozymes corresponding to its metabolic needs, much of which is a result of the continuous exposure to dopamine and norepinephrine from nerve terminals and circulating epinephrine from the adrenal glands (Kaludercic et al., 2011, 2014). A recent study completed in our laboratory showed that MAO activity is highly variable between individuals and that it is a major generator of ROS in human atrial myocardium (Anderson et al., 2014a).

MAO has both direct and indirect ROS-producing capacity. It has been shown that in isolated brain mitochondria, MAO directly generates a 48-fold greater rate of H_2_O_2_ than the ETS (Hauptmann et al., 1996). The H_2_O_2_ produced is converted into water and molecular oxygen by peroxidases, and the aldehyde metabolite produced is efficiently converted by aldehyde dehydrogenase (ALDH) into its respective carboxylic acid. In mitochondria, ALDH2 has been shown to be responsible for converting the highly reactive aldehyde metabolites of dopamine oxidation (3,4-dihydroxyphenylacetaldehyde, DOPAL), and serotonin (5-hydroxyindole-3-acetaldehyde, 5-HIAL) into their corresponding acetates (Ambroziak and Pietruszko, 1991; Rooke et al., 2000). When MAO activity is high, a surge in catecholamine levels would be expected to cause greater ROS production from the ETS, suppressed ATP production, insufficient calcium handling, and mitochondrial DNA damage due to formation of these reactive byproducts if there is not an appropriate antioxidant buffering capacity present.

In addition to mitochondrial sources, the enzyme NADPH oxidase (NOX) is a major producer of ROS in striated muscle. NOX enzyme expression has been documented in the heart at various stages of development, with NOX4 isoform predominating in early developmental stage (Li et al., 2006), and NOX2 in the adult heart (Ambroziak and Pietruszko, 1991). Sakellariou and co-workers recently showed that NOX activity constituted the bulk of ROS production in skeletal myofibers at rest and during contraction, corresponding to expression of NOX2 and NOX4 isoforms in this tissue (Sakellariou et al., 2013).

#### Lipid peroxidation—a unique and important ROS

The mitochondrial membranes contain a high density of polyunsaturated fatty acids (PUFAs) and phospholipids which are susceptible to peroxidation (Bacot et al., 2003; Osman et al., 2011). Phosphatidylethanolamine and phosphatidylcholine are the most abundant phospholipids and comprise ~40% and ~ 30% of total mitochondrial phospholipids, respectively. Cardiolipin and phosphatidylinositol (PI) account for ~ 10–15% of phospholipids, whereas phosphatidic acid (PA) and phosphatidylserine (PS) comprise ~ 5% of the total mitochondrial phospholipids. Cardiolipin is concentrated in the inner mitochondrial membrane in close proximity to the ETS. Although it comprises a relatively a low percentage of total membrane phospholipid, its peroxidation is directly associated with a decline in activity of respiratory complexes III and IV, and the release of cytochrome c which initiates apoptotic signaling (Fry and Green, 1981; Paradies et al., 1999; Chicco and Sparagna, 2007). This is significant because cardiolipin is especially susceptible to peroxidation.

Upon formation, lipid peroxides either propagate to form more lipid peroxides, or spontaneously degenerate to form more stable lipid peroxide products (LPPs) (Bridges et al., 1983; Esterbauer et al., 1991). The final LPP formed is dependent on the initial fatty acid oxidized. One particularly reactive LPP formed via peroxidation of n-6 PUFAs such as arachidonic acid (AA, illustrated in Figure 2) is the aldehyde 4-Hydroxynonenal (HNE). This is particularly relevant because the Western diet contains a high ratio of n-6 to n-3 PUFAs, ~10:1 by some estimates (Guichardant et al., 2004; Cordain et al., 2005). HNE is relatively stable, electrophilic and lipophilic thus is capable of traversing across cellular compartments. Approximately 2–8% of HNE formed reacts with proteins as well as DNA- guanine residues to form HNE-adducts (Nair et al., 1999; Siems and Grune, 2003; Blair, 2008; LoPachin et al., 2009; Minko et al., 2009; Maier et al., 2010; Chavez et al., 2011).

### Deleterious (i.e., maladaptive) consequences of persistent oxidative stress with cardio-metabolic diseases

The presence of a persistent sub-lethal oxidative stress in striated muscle has been associated with a variety of cardio-metabolic diseases for many years. Recent studies have uncovered potential roles for oxidative stress as causal factors underlying these diseases, although extracting clearly defined roles for disease causality vs. simple association remains a challenge. Due to its primary function in regulating cellular metabolism and oxygen consumption, the mitochondrial ETS is known to increase ROS production in striated muscle with a wide variety of diseases involving metabolic supply-demand mismatch. In experimental and clinical models of obesity/type 2 diabetes (O/T2D), an increase in ROS production from mitochondrial ETS has been reported in both heart (Boudina and Abel, 2007; Anderson et al., 2009, 2012; Boudina et al., 2009) and skeletal muscle. During ischemia, mitochondrial ROS is increased from several sites, largely a result of oxidative damage to ETS and to enzymes in the matrix such as aconitase. Upon reperfusion following ischemia, a burst of ROS from the ETS is a well-characterized phenomenon that has been implicated to be both beneficial under some contexts (e.g., pre-conditioning, see next section), or deleterious. Comprehensive reviews of current knowledge regarding the sources and consequences of mitochondrial ROS during ischemia and reperfusion in striated muscle can be found elsewhere (Tullio et al., 2013; Chen et al., 2014; Lejay et al., 2014).

MAO's unique orientation relative to the ETC provides the ability for MAO to interfere with mitochondrial function and cellular energetics, although very little is known about the potential contribution of MAO-derived ROS in cardio-metabolic diseases. One study reported that MAO-derived ROS led to diminished state 3 and state 5 respiration (Cohen and Kesler, 1999). Furthermore, Hauptmann and colleagues showed that MAO-derived H_2_O_2_ can introduce mutations in mitochondrial DNA (mtDNA). Their results demonstrated that H_2_O_2_, produced from MAO exposure to tyramine, can readily diffuse through the inner mitochondrial membrane and produce a significant increase in mtDNA single strand breaks (Hauptmann et al., 1996). Because of MAO's proximity to the mitochondrial oxidative phosphorylation system, ROS produced by MAO could conceivably disrupt the ATP-dependent electrochemical and contractile functions of cardiomyocytes, given the heavy reliance on mitochondria for ATP in these cells (Berman and Hastings, 1999; Kaludercic et al., 2011).

In addition to altering mitochondrial energetics, the byproducts of MAO deamination can wreak havoc on the cell by stimulating apoptotic and hypertrophic pathways, and furthermore may trigger other sites within the cell to release ROS. In myocardium, MAO-derived H_2_O_2_ at low levels can stimulate hypertrophic and fibroblast proliferative pathways, including ERK1/2 (Sabri et al., 1998) and NFAT 3/4 (Kaludercic et al., 2010) while higher levels lead to apoptotic and necrotic pathways via Bax up-regulation (Bianchi et al., 2005). Aldehyde metabolites may initiate lipid peroxidation which can increase levels of 4-HNE (Kaludercic et al., 2010), inactivate proteasomes (Farout and Friguet, 2006) and the ETS (Choksi et al., 2004) as well. Recent studies have specifically investigated the role of MAO and ROS production in the etiology of cardiac disease since cardiomyocytes are constantly exposed to catecholamines *in vivo*. Kaludercic and colleagues studied the role of MAO-derived ROS in the development of cardiac hypertrophy induced with pressure overload through transverse aortic constriction (TAC). The results of these studies showed that compared to MAO-B null mice, wild-type mice showed a significantly greater increase in left ventricle size, decreased fractional shortening, and increased levels of HNE and fibrosis following TAC, signifying higher ROS formation and lipid peroxidation due to MAO activity (Kaludercic et al., 2014). Similarly, studies in a mouse model of diabetes revealed that MAO-A activity and mitochondrial mass in hearts of diabetic mice were higher than in normo-glycemic mice. The researchers hypothesized that insulin resistance had led to the diminished antioxidant capacity of the cell, in turn leading to more susceptibility to MAO-derived ROS, further illustrating the connection between nutrient overload and diminished antioxidant capacity (Mellor et al., 2010; Manni et al., 2013).

The pathophysiologic involvement of ROS derived from NOX activity in striated muscle remains an area of active investigation. NOX-derived ROS has been shown to be increased and associated with mitochondrial dysfunction and insulin resistance in both heart (Vazquez-Medina et al., 2013) and skeletal muscle (Yokota et al., 2009) of obese rodent models. Other reports clearly implicate myocardial NOX involvement in the development of cardiac hypertrophy and fibrosis in O/T2D (Yamamoto et al., 2008; Fukuda et al., 2010), pressure overload and heart failure (Bendall et al., 2002; Byrne et al., 2003; Heymes et al., 2003; Johar et al., 2006). With myocardial ischemia, however, the role of NOX-derived ROS is ambiguous. Despite evidence that NOX expression and activity is induced in experimental (Meischl et al., 2006) and clinical studies (Krijnen et al., 2003) of myocardial ischemia, myocardial NOX2- and p47^PHOX^-deficient mice exhibit no changes in infarct size compared to wild-type following ischemia/reperfusion injury (Hoffmeyer et al., 2000; Frantz et al., 2006). Recent studies have shed some light on these disparate findings. An intriguing study by Matsushima and co-workers showed that myocardial infarct size/area at risk was substantially higher in mice when both systemic NOX2 and myocardial NOX4 was absent, and they attributed this to a downregulation of adaptive response pathways mediated by hypoxia-inducible factor-1α (HIF1α) in the hearts of these double-knockout mice (Matsushima et al., 2013). Another recent study provided very compelling evidence that NOX4 activity is absolutely necessary for maintaining optimal cardiac redox environment under normoxic conditions, and that eliminating cardiac NOX4 promoted reductive stress and exacerbated ischemia/reperfusion injury in mice (Yu et al., 2014). These studies, and others, illustrate the highly complex roles for NOX in the heart, demanding that further investigation of ROS production is necessary to understand the extent to which perturbations of the redox environment underlie cardio-metabolic diseases.

Until recently, LPPs have been viewed as mere bi- products and bio-markers of oxidative stress (Gutteridge, 1995; Niki, 2009). A rapidly growing body of scientific literature now places LPPs at the front of pathophysiology for a large number of diseases involving striated muscle. In a toxicological study of neonatal cardiomyocytes, HNE caused apoptosis associated with a decrease in GSH, a surge in intracellular Ca^2+^ levels and a loss of mitochondrial membrane potential (Hortigon-Vinagre and Henao, 2014). In skeletal muscle of the diaphragm, protein carbonylation derived from LPPs has been shown to be increased in sepsis, mechanical ventilation and COPD and this contributed to muscle contractile dysfunction in these conditions (Barreiro, 2014).

Several groups have reported increased lipid peroxidation and carbonyl stress in the cardiovascular system of diabetic patients (Arzamastseva et al., 2007; Anderson et al., 2009; Annadurai et al., 2014). Studies in diabetic rat heart show that HNE binds preferentially to cysteine > histidine > lysine residues in the mitochondria (LoPachin et al., 2009). Residues that become modified in this way are often found in the active-site of enzymes, and also in structural motifs responsible for maintaining tertiary and quaternary structure (Korotchkina et al., 2001; Levonen et al., 2004). Mitochondrial protein modification by HNE corresponds to reduced enzyme activity for key enzymes such as adenine nucleotide translocase (ANT), succinate dehydrogenase and α-ketoglutarate dehydrogase in the hearts of diabetic rats (Chen et al., 1995, 2001; Benderdour et al., 2003; Nguyen and Picklo, 2003; Lashin et al., 2006; Chavez et al., 2011). HNE modification of mitochondrial enzymes is represented by yellow lightning bolts shown in Figure 2.

### Compensatory mechanisms for adaptation to LPPs and carbonyl stress

One of the exquisite features of striated muscle, particularly the heart, is that it is very plastic and can adapt to a broad array of stressors. Carbonyl stress arising from PUFA peroxidation is known to activate genes involved in phase I and II antioxidant/detoxification, mitochondrial biogenesis, and other pathways, all of which serve to restrain the further production of PUFA-derived aldehydes and suppress their toxic effects. A primary adaptive response pathway that is activated by oxidative and carbonyl stress involves NF E2-related factor-2 (Nrf2). Electrophiles such as LPPs and PUFA-derived aldehydes generated during periods of oxidative stress activate Nrf2 by liberating it from its tethering protein Keap1 in the cytosol, allowing it to translocate to the nucleus where it binds to antioxidant/anti-inflammatory response elements (AREs) in promoter regions of genes/proteins involved in glutathione synthesis and phase II detoxification (Motohashi and Yamamoto, 2004). Studies have shown that HNE induces nuclear accumulation of Nrf2 and up-regulates many GSH-synthesizing enzymes and proteins (reviewed in Chapple et al., 2013). Indeed, pre-conditioning of the heart by retro-orbital injection of HNE induces cardioprotection from ischemia/reperfusion injury via Nrf2, and this cardioprotection is lost in Nrf2^−/−^ mice (Zhang et al., 2010). The roles and regulation of Nrf2, and other transcription factors that activate the ARE, is well beyond the scope of this review, and the reader is referred to other excellent review articles on this topic (Sykiotis and Bohmann, 2010; Tkachev et al., 2011; Chartoumpekis and Kensler, 2013).

In addition to Nrf2, recent evidence has implicated members of the peroxisome proliferator activated receptor (PPAR) family in the cellular response to ROS. Several reports have documented a key role for PPARα and PPARγ in augmenting the antioxidant capacity of liver (Toyama et al., 2004; Abdelmegeed et al., 2009; Guelzim et al., 2011) and vascular tissue (Inoue et al., 2001a,b) in response to oxidized PUFAs. Our group recently completed a small clinical trial and observed that a concentrated dose of fish oil n-3 PUFA's was associated with up-regulation of several antioxidant enzymes, particularly mitochondrial-localized thioredoxin reductase-2 (TxnRd2), and this was accompanied by increased nuclear transactivation of PPARγ, in atrial myocardium of patients undergoing cardiac surgery (Anderson et al., 2014b). This finding is particularly intriguing given the emerging evidence that TxnRd2 is critical for maintaining mitochondrial redox balance in multiple cell types and tissues, including the heart (Rigobello et al., 2005; Stanley et al., 2011; Fisher-Wellman et al., 2013). These articles and other recent literature documenting the beneficial adaptations to ROS in striated muscle under various physiological contexts are listed in Table 1.

**Table 1:** **Recent literature illustrating beneficial adaptations to ROS in striated muscle**.

**Muscle**	**Physiological state/disease**	**References**
Myocardium	Ischemia/reperfusion	Kevin et al., 2003a; Dost et al., 2008; Liu et al., 2008; Zhang et al., 2010; Penna et al., 2013; Tullio et al., 2013; Kalogeris et al., 2014
Myocardium	Exercise	Akita et al., 2007; Sanchez et al., 2008; Nelson et al., 2011; Frasier et al., 2013; Fittipaldi et al., 2014
Myocardium	Nutrient overload	Joyeux-Faure et al., 2006; Anderson et al., 2012; Tocchetti et al., 2012; Fisher-Wellman et al., 2013; Rindler et al., 2013; Chen et al., 2014; Lejay et al., 2014.
Skeletal muscle	Exercise	Gomez-Cabrera et al., 2005, 2008; Kang et al., 2009; Ristow et al., 2009; Powers et al., 2010; Strobel et al., 2011; Powers et al., 2011b; Fisher-Wellman et al., 2013; Radak et al., 2013; Ferraro et al., 2014

Another key adaptive response to ROS includes activation of cellular quality control pathways in myocytes, namely autophagy and mitophagy. These pathways are a very active and rapidly expanding area of research, and well beyond the scope of this review. The role of autophagosome formation in the adaptive response to ROS in myocytes are shown in Figure 2 and is the topic of a number of thorough reviews (Gurusamy and Das, 2009; Lee et al., 2012c; Rahman et al., 2014).

LPP detoxification occurs at two main stages. The first is at the site of lipid peroxidation itself (membrane-localized), and the second is in the cytosol. Following their formation, LPPs may undergo phase I biotransformation where they are conjugated to antioxidant molecules (i.e., glutathione). Alternatively, LPPs are converted by phase II detoxification enzymes such as aldehyde dehydrogenase (Budas et al., 2009), which converts LPPs to their corresponding alcohols. Below we discuss some of the major enzymes involves in the detoxification of LPP-derived aldehydes. It is important to note for many of these enzymes, their enzymatic efficiency is itself decreased by aldehydes which may be key factor in the limitation of antioxidant capacity. A list of the major enzymes responsible for detoxifying LPPs, along with the reaction catalyzed by the enzyme, can be viewed in Box 1.

Box 1 **Mechanism of LPP clearance by different enzyme systems.**

Glutathione dependent enzymes

1. Glutathione Peroxidase 4
  ▪ ROOH + 2GSH → GSSG + ROH + H2O
2. Glutathione S-transferase (GST)
  ▪ ROOH + GSH → ROH + GSSG + H_2_0

Aldehyde Oxidizing enzymes

3. Aldehyde dehydrogenases
  ▪ RCHO + NAD+ + H_2_O → RCOOH + NADH + H+ Molybdenum hydroxylases
4. Aldehyde oxidase (Pryde et al., 2010)
  ▪ aldehyde + H_2_O + O_2_ → a carboxylic acid + H_2_O_2_
5. Xanthine Oxidase
  ▪ Xanthine + H_2_O + NAD+ ←===→ urate + NADH
  ▪ Hypoxanthine + H2O + NAD+ ←===→ xanthine + NADH
  ▪ Xanthine + H_2_O + O2 ←===→ urate + H2O2

Aldehyde reducing enzymes

  ▪ Typical reaction: RCHO + NAD+ + H2O → RCOOH + NADH + H+
6. Alcohol reductase
7. Carbonyl reductases
8. Aldo-ketoreductases

#### Glutathione-dependent enzymes

***Glutathione peroxidase 4***. GPX4 is a member of the glutathione peroxide family of selenoenzymes enzymes. In addition to being able to neutralize small hydroperoxides such as H_2_O_2_, it is one of only a few enzymes capable of neutralizing complex and bulky hydroperoxides (e.g., cholesterol hydroperoxides) (Brigelius-Flohe, 1999; Imai et al., 2003; Yant et al., 2003; Brigelius-Flohe and Maiorino, 2012). GPX4 is unique in that unlike other GPXs, it is not limited to glutathione as a substrate (Brigelius-Flohe and Maiorino, 2012). Its protein thiols can perform the function of GSH, and as a result, GPX4 may function as either a glutathione peroxidase or a thiol peroxidase adapting to perform its functions depending on cellular redox conditions.

There are 3 major isoforms of GPX4: cytosolic, mitochondrial and nuclear (Regev-Rudzki and Pines, 2007). In the mitochondria, GPX4 over-expression has been shown to protect against cytochrome c release, generation of H_2_O_2_ and loss of membrane potential following ischemia/reperfusion injury (Imai et al., 2003). In diabetic mice, over expression of mitochondrial GPX4 was protective against cardiac ischemia reperfusion injury and preserves mitochondrial integrity (Chen et al., 2001; Hollander et al., 2003; Liang et al., 2007; Dabkowski et al., 2008).

***Glutathione S-transferase (GST)***. The GSTs conjugate electrophilic aldehydes with GSH to yield a less reactive conjugate that is eliminated from the cell by glutathione S-conjugate efflux pumps, this process not only increases the solubility of conjugated compounds but marks these structures for elimination from the cell (Hayes and Pulford, 1995). ROS regulate the expression of GST via c- fos and c jun signaling (Daniel, 1993). A study in rat hepatocytes reported that for HNE, metabolism by GST is responsible for the majority of HNE removal (~60%); a little is metabolized by the alcohol and aldehyde dehydrogenases (10%) leaving ~24% of 4-HNE clearance for other mechanisms (Hartley et al., 1995).

#### Aldehyde-metabolizing enzymes:

***Aldehyde dehydrogenase***. The ALDHs are particularly relevant in the mitochondria as metabolizers of aldehydes generated from PUFAs and monoamine oxidase, as it has been demonstrated that inhibiting ALDH activity has a direct impact on the metabolism of amines by MAO (Ambroziak and Pietruszko, 1991; Rooke et al., 2000). There are 19 known isoforms of the ALDH enzyme with ALDH2 as the major mitochondrial isoform (Marchitti et al., 2008). In human populations, individuals with a polymorphism for ALDH2 had higher levels of the biomarker C-reactive protein following myocardial infarction (Bian et al., 2010). In the SAPPHIRe prospective cohort study, patients with ALDH2 genetic variants with a possessed two-fold greater risk of progression to clinical hypertension (Chang et al., 2012). ALDH3A2 also was found to be important in redox-related pathologies stemming from nutritional overload. It is expressed in both the heart and skeletal muscle and displays a substrate specificity for aldehydes of various long and short chain fatty acids.

***Molybdenum hydroxylases***. There are two main enzymes in this class (1) Aldehyde oxidase (AOX): This enzyme oxidizes aldehydes to carboxylic acids and it is relatively nonspecific and its endogenous role is not well understood. (2) Xanthine Oxidase: exists as xanthine dehydrogenase-metabolism of aldehydes by xanthine oxidase generates radicals. These two enzymes share some amino acid sequence homology and both contain molybdenum and iron as well as flavin adenine dinucleotide (FAD) as co-factors.

***Carbonyl reductases***. Carbonyl reductases are part of short chain dehydrogenases/reductases (SDR) family of proteins. These enzymes are ubiquitous and are largely localized to the cytosol with some isoforms in the mitochondria and peroxisomes (Rosemond and Walsh, 2004).

***Aldo-ketoreductases***. The aldose reductases metabolize lipid aldehydes and their glutathione conjugates (Srivastava et al., 2003). AKR1 specifically metabolizes aldehydes produced by MAO using NADPH. The mitochondrial isoform accounts for 5% of cell activity and metabolizes 4-HNE. AKR1B uses both NADH and NADPH and is also localized in the cytosolic fraction (Srivastava et al., 1999). It is highly expressed in the cardiovascular system, appears to be partly responsible for reducing aldehydes such as 4-HNE, and its activity is potentiated when the substrate is conjugated to glutathione.

## Role of ROS in cardiac preconditioning

**Figure 3:**
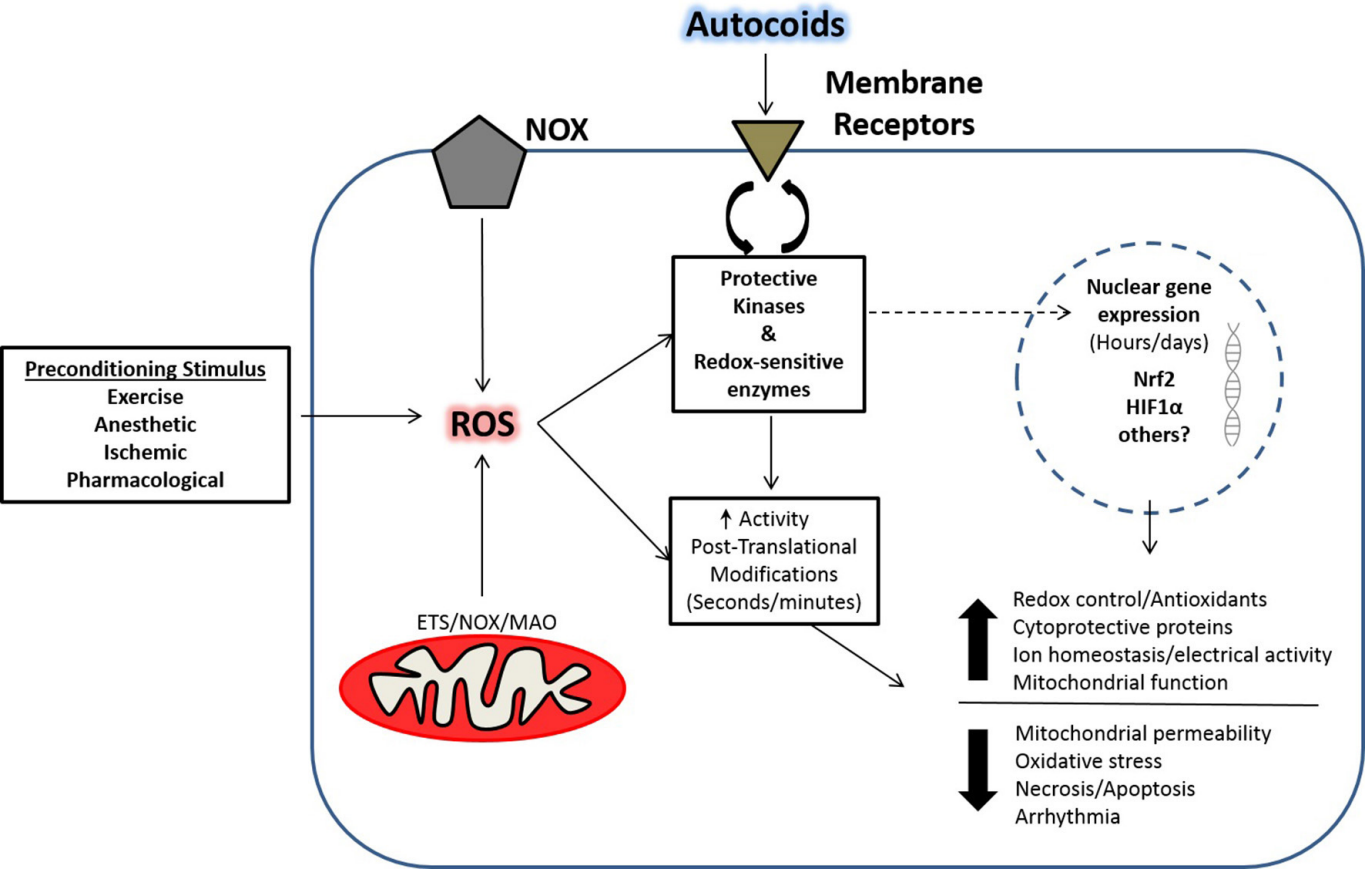
**Pre-conditioning and ROS-mediated effects in striated muscle**. Shown is a schematic illustrating the development of ROS-mediated adaptations from various preconditioning paradigms as described in the text. Adaptive responses that lead to preconditioning in cardiac muscle culminate in protection through early post-translational modifications to redox sensitive enzymes, and late adaptations through enhanced translocation of key transcription factors. The ensuing adaptations result in the early- and late-window of preconditioning that have been described in the cardioprotection literature. (ROS, Reactive oxygen species; NOX, NADPH-oxidase; ETS, Electron transport system; MAO, Monoamine oxidase; Nrf2, NF E2-related factor-2; HIF1α, Hypoxia-inducible factor-1α; Autocoids, factors secreted from cells with paracrine-like effects).

The role of ROS in cardiac health and disease has become increasingly recognized in research as a part of normal physiology and pathophysiology due to the multitude of cell regulatory layers associated with redox biology. Although once thought of as strictly deleterious to cardiovascular function, the production of ROS can also elicit an adaptive cardiac preconditioning response. These beneficial ROS are thought to set in motion signaling events that can elicit alterations in gene expression, as well as acute events that can activate proteins in a post-translational manner. In this regard, several stimuli have been shown to evoke cardioprotection in a ROS-dependent manner such as volatile anesthetics, ischemic preconditioning, and pharmacological compounds. A number of major ROS-mediated cellular pathways activated in response to these stimuli are illustrated in Figure 3.

### Anesthetic preconditioning

Cardiac preconditioning with volatile anesthetics has received a considerable amount of attention as a strategy to mitigate I/R injury through their negative inotropic, anti-arrhythmic and myocardial infarct salvaging effects (Sahlman et al., 1995; Cope et al., 1997; Schlack et al., 1997; Lamberts et al., 2009; Riha et al., 2011; Wojtovich et al., 2011). Several reviews have highlighted mechanisms involved in anesthetic-induced cardioprotection, and ROS have been commonly identified as an important element to the protective mechanism (Hu and Liu, 2009; Bonney et al., 2014; Swyers et al., 2014). ROS are known to be intricately involved in post-ischemic myocardial dysfunction and infarction, and a reduction in oxidative stress has been identified as an important component in alleviating I/R injury; (Hamilton et al., 2004; Dabkowski et al., 2008). Paradoxically, transient bursts of ROS during anesthetic treatment plays an important role in the cardioprotective response (Mullenheim et al., 2002; Novalija et al., 2002; Tanaka et al., 2003). In line with these concepts, treatment with isoflurane (Tanaka et al., 2002, 2003) or sevoflurane (Kevin et al., 2003b) increases ROS production during preconditioning, and reduces oxidative stress during I/R (Nakamura et al., 1999; Kevin et al., 2003b). Following anesthetic exposure, an increase in intracellular superoxide production can be detected by the fluorescence of oxidized dihydroethidium (Tanaka et al., 2002, 2003; Ludwig et al., 2004). This transient ROS burst is important for the preconditioning phenotype as antioxidant treatment negates the beneficial effects. In particular, isoflurane-induced infarct salvage is abolished when bracketing anesthetic administration with intravenous infusion of various antioxidants. These antioxidants include: N-2-mercaptoproprionyl glycine (MPG), Mn(III) tetrakis(4-benzoic acid) porphyrine chloride (MnTBAP), mitotempol, or the glutathione precursor N-acetylcysteine (NAC) (Mullenheim et al., 2002; Tanaka et al., 2003; Hirata et al., 2011). Similarly, the infarct salvaging effect of sevoflurane is abolished by various ROS scavengers such as glutathione, superoxide dismutase, catalase, MPG or MnTBAP (Novalija et al., 2002; Kevin et al., 2003b; Lamberts et al., 2009). Studies comparing desflurane and sevoflurane have demonstrated similar mechanisms of protection during a metabolic insult (Hanouz et al., 2007; Sedlic et al., 2009). *In vitro* studies have also corroborated the importance of ROS in anesthetic preconditioning. One study demonstrated that human right atrial contraction during hypoxia/reoxygenation was better preserved following treatment with desflurane and sevoflurane, and the protective benefits were lost with administration of MPG (Hanouz et al., 2007). Another study demonstrated that desflurane and sevoflurane protected rat isolated left ventricular cells against H_2_O_2_ induced cell death, and the antioxidant trolox abolished protection. Interestingly, ROS production by desflurane was higher than sevoflurane during preconditioning, and this translated to greater myocyte survival (Sedlic et al., 2009). Taken together these studies provide a compelling argument that transient ROS production acts by influencing downstream cardioprotective signaling events. The mechanism of ROS release as a protective stimuli in anesthetic-induced preconditioning has also been linked to the protection observed with the ischemic preconditioning (IPC) phenomenon, suggesting that these two regimens have commonalities in their cardioprotective mechanisms (Swyers et al., 2014). Indeed IPC also has a strong literature implicating ROS as a triggering mechanism in cardioprotection.

### Ischemic preconditioning

The discovery of IPC by Murry et al., in 1986 has paved the way for an ample amount of research dedicated toward unveiling underlying mechanisms of cardioprotection. IPC is accomplished through short intermittent bouts of I/R, typically one to five cycles, that leads to endogenous protection against an ensuing longer duration ischemia (Murry et al., 1986). Since then, several pathways leading to cardioprotection have been proposed, but a central theme that has been a mainstay in the IPC literature is that ROS play an intricate role in the cardioprotective signaling mechanisms (Shiomi et al., 2013). The beneficial effects of IPC include a reduction in myocardial infarction (Murry et al., 1986), reperfusion arrhythmias (Shiki and Hearse, 1987), stunning (Bolli et al., 1997), and necrotic and apoptotic cell death (Nakamura et al., 2000). A beneficial role for ROS in IPC is now well-established in the protective signaling cascade. Exposure to a single bout of IPC reduces infarct size, and cardioprotection is abolished when various free-radical scavengers are administered before and/or during IPC (Tanaka et al., 1994; Baines et al., 1997; Dost et al., 2008; Liu et al., 2008). Findings on the time frame of ROS production during preconditioning has been equivocal with evidence that the beneficial ROS are generated during the ischemic phase (Kevin et al., 2003a), and others showing the reperfusion phase (Dost et al., 2008). It has been documented by multiple groups that a significant amount of ROS can be generated by mitochondria during ischemia (Vanden Hoek et al., 1998; Becker et al., 1999; Kevin et al., 2003a), which can be blunted with ETS inhibitors for sites I and III. It is also known that there are overlapping mechanisms involved in IPC (Xin et al., 2012). This is evident by studies demonstrating that free-radical scavengers do not abolish IPC-mediated cardioprotection when multiple preconditioning cycles are used (Iwamoto et al., 1991). The preconditioning mechanism is multifactorial (Yang et al., 2010), so the use of multiple cycles may heighten signaling cascades through other pathways irrespective of ROS. The precise mechanisms on how IPC-induced ROS mediate cardioprotection are still debated, and more research is needed to verify spatial and temporal activation in the cell, as well as end effectors. The use of pharmacological agents that that mimic IPC in a laboratory setting may ultimately foster identification of novel therapeutic approaches.

### Pharmacological preconditioning

There are several pharmacological approaches that have been shown to mimic the preconditioning phenomenon. Diazoxide is a potassium channel activator whose cardioprotective characteristics are thought to derive from a ROS preconditioning stimuli leading to activation of downstream end-effectors (Garlid et al., 1997; Tritto et al., 1997; Pain et al., 2000; Forbes et al., 2001). This is supported by studies where the antioxidants MPG and NAC reduced diazoxide-induced ROS production, and block the recovery of LVDP following global I/R (Forbes et al., 2001). Although cardiomyocytes contain both a mitoK_ATP_ channel and a sarcolemmal K_ATP_ channel, diazoxide and the potassium channel blocker 5-Hydroxydecanoate (5-HD) are thought to have a higher specificity for the mitoK_ATP_ channel (Garlid et al., 1996; Yellon and Downey, 2003). In addition, other potassium channel openers nicorandil and cromakalim have been shown to increase superoxide production in isolated hearts, and the mitoK_ATP_ channel blockers 5-HD and glibenclamide decreased ROS production (Obata and Yamanaka, 2000). Follow up studies show that nicorandil protects against *in vivo* I/R injury in rabbit hearts; and the mitoK_ATP_ channel blocker 5-HD abolished protection, while blockade of the sarcolemmal K_ATP_ channel had no effect (Das and Sarkar, 2003). Importantly, treatment with nicorandil during the reperfusion period did not provide protection, which supports the idea that apriori ROS are setting the stage as a trigger for protective signaling cascades. Others studies investigating the cardioprotective mechanism of acetylcholine and bradykinin demonstrate that an increase in ROS production during treatment is a central component to their infarct sparing effects because the antioxidant MPG abolishes protection (Cohen et al., 2001; Krieg et al., 2004). The authors concluded that agents signaling through G_i_ proteins share common preconditioning pathways, but adenosine induced protection diverts from this hypothesis, because an adenosine receptor agonist co-administered with MPG or 5-HD did not abolish protection (Cohen et al., 2001). Although adenosine receptor activation can reduce infarct size irrespective of ROS signaling, the downstream preconditioning cascade may be similar in regard to intermediate signaling through mitoK_ATP_ channels (Miura et al., 2000). Statin treatment has also been implicated in cardioprotection through the upregulation of ROS production by mitoK_ATP_ channel activation. The improved post-ischemic contractile function observed with parvastatin was abolished with NAC and 5-HD. The authors concluded that parvastatin leads to the activation of mitoK_ATP_ channels and that downstream activation of PKC mediates the protective phenotype. However, the order of events in this signaling cascade is still under investigation. Mild mitochondrial uncoupling has also been implicated as a preconditioning mechanism, although irrespective of mitoK_ATP_ channel activation (Holmuhamedov et al., 1999). Support for a role of ROS release during uncoupling comes from studies where low concentrations of p-(trifluoromethoxy)phenylhydrazone (FCCP) improved post-ischemic mechanical function. These studies demonstrate that FCCP increased ROS independent of mitoK_ATP_ channel activation. Importantly protection was lost with antioxidant administration, but not with mitoK_ATP_ channel blockers (Brennan et al., 2006). Caveats to studies implicating mitoK_ATP_ channels in preconditioning are the use of diazoxide and 5-HD, which has been shown to have non-specific effects (Hanley et al., 2003; Drose et al., 2006; Li et al., 2010). In addition, there is still uncertainty as to the function and existence of mitoK_ATP_ channels due to the elusiveness of their molecular composition (reviewed in Szabo and Zoratti, 2014). A recent study using a comprehensive proteomics analysis of mitochondrial inner membrane proteins combined with RNA knockdown technique suggested that *KCNJ1*-encoded renal outer medullary potassium channel (ROMK) is a critical pore-forming subunit of the mitoK_ATP_ channel (Foster et al., 2012). Clearly, more studies using targeted molecular approaches such as this are needed to precisely identify all components of this potentially very important channel.

### Sources of ROS: where do they come from?

Many of the preconditioning studies reviewed thus far suggest that beneficial ROS production from various stimuli are mitochondrial in origin (Baines et al., 1997; Vanden Hoek et al., 1998; Das et al., 1999; Cohen et al., 2001; Tanaka et al., 2002; Andrukhiv et al., 2006; Sedlic et al., 2009). There has been an ample amount of research showing that the opening of mitochondrial channels/pores and fluctuations in mitochondrial membrane potential leads to the release of ROS (O'Rourke et al., 2007; Aon et al., 2010; Brown and O'Rourke, 2010; Gauthier et al., 2013), but whether channel/pore opening acts as a trigger vs. a mediator of cardioprotection is still being debated. While it is known that ROS are a commonality to IPC, controversy exists as to when the ROS are released. In isolated chick cardiomyocytes exposed to hypoxic preconditioning an increase in ROS production was observed during the hypoxic period that is abrogated with the complex III inhibitor myxothiazol, but not a NOX or NOS inhibitor (Vanden Hoek et al., 1998). Although ROS have been shown to be released during the ischemic cycle of IPC (Vanden Hoek et al., 1998; Kevin et al., 2003a), Dost et al. propose that ROS generated during the reperfusion phase are providing the redox signals for cardioprotection because MPG administered during the ischemic cycle did not abolish the infarct salvaging effect of IPC (Dost et al., 2008). More recent studies implicate mitochondrial derived ROS, nitric oxide, and the mitochondria redox state as possible regulators of mitoK_ATP_ channel opening in IPC-mediated cardioprotection (Queliconi et al., 2011). Pharmacological activation of the mitoK_ATP_ channel is presumed to proceed in a similar manner to that of IPC, however, as mentioned previously, the relationship between PKC activation, ROS production, and mitoK_ATP_ channel activation is still being investigated (Tritto et al., 1997; Pain et al., 2000; Liu et al., 2008; Yang et al., 2010). The mechanism of ROS release following mitoK_ATP_ channel opening is thought to occur due to mitochondrial uptake of potassium leading to slight alkalization and mild uncoupling (Andrukhiv et al., 2006), however the precise mechanism is unknown. Further evidence using the mitoK_ATP_ channel openers diazoxide or cromakalim suggests that ROS are derived from site I of the ETS because rotenone did not have an effect on myxothiazol-induced ROS production (Andrukhiv et al., 2006). Also, complex III of the ETS has been shown to generate ROS during IPC (Vanden Hoek et al., 1998); and similar findings were reported when mitoK_ATP_ channels were activated through bradykinin-induced preconditioning (Oldenburg et al., 2004). Furthermore, complex III generated ROS appear to also mediate preconditioning with isoflurane because myxothiazol treatment abolished the ROS burst as well as the infarct salvaging effects of isoflurane (Ludwig et al., 2004). Taken together, these findings suggest that mitochondrial derived ROS, particularly from the ETS, are responsible for a large portion of ROS release in models of preconditioning. This further supports the idea of an optimal “zone” where ROS release results in favorable adaptions, whereas exacerbated ROS release can lead to maladaptive responses in diseased states.

### End-effector mechanisms: what leads to protection?

If ROS are a playing a substantial role in cardioprotection the question becomes, what are the end-effector mechanisms? In models of IPC there are several hypothesis implicating ROS as a means to an end-effector response. An increase in ROS production during IPC is proposed to activate an inner mitochondrial PKC-ε that phosphorylates and inhibits mitochondrial permeability transition (Costa et al., 2006; Costa and Garlid, 2008). A higher threshold for mitochondrial permeability transition following IPC has been demonstrated by several groups and is thought to be one of the mechanisms by which IPC reduces I/R injury (Argaud et al., 2004; Costa et al., 2006; Clarke et al., 2008). Studies have shown that PKC-ε interacts with mitochondrial proteins that constitute the transition pore, and that PKC-ε can phosphorylate the mitochondrial voltage-dependent anion channel (VDAC) *in vitro* (Baines et al., 2003). However, others have failed to observe a change in phosphorylation of mitochondrial permeability transition pore components following IPC (Clarke et al., 2008). This study did observe that IPC inhibited mitochondria permeability transition, but attributed it to a reduction in oxidative stress during I/R injury; although they did not rule out PKC-ε as part of the protective signaling mechanism. Further support for the importance of PKC is provided by studies demonstrating that preconditioning with diazoxide can be blocked when the PKC inhibitor chelerythrine is administered either before diazoxide pretreatment or 10 min before I/R (Takashi et al., 1999), suggesting that the activation of a PKC-ε isoform is playing a downstream role in this form of cardioprotection. However, others hypothesize that PKC activation during IPC sensitizes the myocardium to adenosine, leading to the activation of reperfusion injury salvage kinases such as Akt and ERK (Kuno et al., 2008; Yang et al., 2010).

## Exercise-dependent adaptations evoked by ROS

The relationship between an active lifestyle through physical activity/exercise with increased longevity and decreased mortality is well known (Hamalainen et al., 1995; Lee et al., 2012a). Although there are numerous adaptive responses following exercise, adaptations at the cellular level present as an increase in skeletal muscle mitochondrial content, which is a hallmark of aerobic exercise training and increased endurance capacity. It has been demonstrated that ROS production is important for this hormetic effect of exercise (Kang et al., 2009), and that co-administration of antioxidants may abolish some of the observed health benefits (Ristow et al., 2009). Therefore, it is of interest to determine what and how adaptations are evoked by exercise-induced ROS.

### Sources of ROS during exercise

Mechanical loading of cardiac (Sanchez et al., 2008; Prosser et al., 2011) and skeletal (Ihlemann et al., 1999; Chambers et al., 2009) muscle during contraction/relaxation has been implicated as a mechanism by which ROS signaling can evoke adaptive responses. Mechanical stretch of mouse extensor digitorum longus (EDL) muscle increases ROS production and promotes enhanced glucose uptake, independent of insulin signaling, and this is abolished with various ROS scavengers (Chambers et al., 2009). An increase in skeletal muscle ROS production during stretch and contraction is evidenced by increases in the fluorescence of the H_2_O_2_ sensitive probe, CM-H_2_ DCF (Sandstrom et al., 2006; Chambers et al., 2009) and GSSG:GSH_T_ levels (Sandstrom et al., 2006), neither of which was observed in the presence of the antioxidant NAC. In line with the idea that intracellular ROS production can influence surrounding tissue, one study demonstrated that local ROS production by contracting skeletal muscle may provide a signal to increase the tissue repair response following exercise. NOX-generated H_2_O_2_ can facilitate the recruitment of neutrophils to skeletal muscle tissue following intense exercise (Nunes-Silva et al., 2014). The manner by which H_2_O_2_ produced this response was through increased intracellular adhesion molecule expression leading to enhanced leukocyte-endothelial interaction within the muscular micovasculature endothelial cells. These findings raise the question as to the locus of ROS production, skeletal muscle vs. vascular, since both tissues are known expressers of the NOX2 isoform (Frey et al., 2009; Barbieri and Sestili, 2012).

A similar role for NOX2 in cardiac tissue has been demonstrated whereby myocardial stretch and tachycardia increases NOX2 generated ROS leading to redox modification of the ryanodine receptor and increased calcium spark frequency (Sanchez et al., 2008; Prosser et al., 2011). Further implications for NOX2 derived ROS in cardiac adaptations to exercise was demonstrated by our laboratory, where inhibition of NOX2 abolished the infarct salvage effect of exercise in rats (Frasier et al., 2013). Indeed other sources of ROS production may be mediating adaptations to exercise as well. Some studies suggest that complex I generated ROS and subsequent release into the cytosol through mitoK_ATP_ channels can evoke cardioprotection (Domenech et al., 2002; Andrukhiv et al., 2006), but the evidence for this as a mechanism of ROS production during exercise is scarce. The phospholipase A2-lipoxygenase pathway has been shown to release ROS into the extracellular space in rat skeletal muscle preps, although the physiological implications for this have not been determined (Zuo et al., 2004). Another factor to take into consideration is the degree and spatial production of ROS as a function of exercise intensity.

During high intensity/exhaustive exercise the production of ROS by xanthine oxidase (XO) appears to increase plasma biomarkers oxidative stress (Vina et al., 2000). An increase in plasma XO activity was shown to be important for the activation of adaptive responses to exercise in skeletal muscle (Gomez-Cabrera et al., 2005), because inhibition of XO with allopurinol prevented the activation of MAPKs and abolished the increase in MnSOD gene expression associated with exercise. However, whether or not these effects were due to ROS generated in the plasma vs. the cytoplasm cannot be inferred. Allopurinol may instead be inhibiting the production of intracellular ROS and preventing activation of redox sensitive pathways, which has been shown to be the case for the activation of PGC-1α following acute exercise (Kang et al., 2009). The role of XO on ROS production has been shown to be miniscule in other studies, where allopurinol did not mitigate ROS production as assessed by GSSG levels. Moreover, XO inhibition did not attenuate exercise-induced mitochondrial biogenesis and antioxidant gene expression with chronic exercise (Wadley et al., 2013), indicating that other ROS producing enzymes may be more important for these adaptations to exercise.

With respect to LPPs formed during exercise, measurements of protein carbonylation have yielded surprising and even contradictory results. One recent study reported that chronic exercise in middle aged human male subjects resulted in increased urinary levels of 8-hydroxy-2′-deoxyguanosine (8-OHdG), 4-HNE carbonyl adducts, and immune system activation, indicative of a greater level of protein carbonylation (Sasaki et al., 2013). However, another study showed that protein carbonylation was decreased in skeletal muscle of rats following an acute bout of swimming (Magherini et al., 2013). This suggests that LPP formation and subsequent protein carbonylation following exercise is complex, and there may be differences in compartmentalization into blood, muscle, and other organs, in addition to time-dependent factors and type of exercise (i.e., acute vs. chronic) to consider. In any case, these findings illustrate the need for a more detailed investigation of the mechanisms and role of LPPs in exercise.

### Mechanisms responsible for ROS-dependent adaptations to exercise

It is now commonly accepted that ROS have both beneficial and detrimental effects on the cell depending on the delicate balance between scavenging and production. There are several fates of ROS that typically play out in a finite span of time due to their relatively short half-life. While it was originally thought that exercising muscle produces ROS that could be detrimental to the cell through the oxidation of macromolecules, the paradigm has shifted over the last several years. However, the mechanism by which ROS exert beneficial cellular adaptations has been hard to identify, and is an area of research that is dynamically evolving. In addition, the production of ROS, or ROS byproducts, by exercising muscle may lead to direct or indirect alterations to the vasculature, or other organs, in an endocrine like manner. However, whether or not exercise-induced adaptations arise from ROS endocrine-like signaling has not been established. In the literature, there are discrepant findings for changes in blood markers of ROS production following different exercise intensities. Recently Bloomer's group did not observe changes in blood H_2_O_2_, malondialdehyde (MDA), or advanced-oxidation protein products immediately after an acute aerobic or high intensity exercise bout in trained men (Bloomer et al., 2005). Similar results have been demonstrated after acute exercise in regard to changes in MDA, although elevated levels of ROS were evident post-exercise as measured by electron spin resonance spectroscopy and an increase in the ratio of oxidized glutathione to total glutathione (GSSG:GSH_T_) (Groussard et al., 2003; Bloomer et al., 2005). These discrepant findings have several implications when taken together: (1) changes in circulating oxidative stress biomarkers may not be indicative of intracellular ROS production; (2) some assays may not be sensitive enough to detect ROS biomarkers; or (3) circulating antioxidant capacity is sufficient to handle the oxidative burden if ROS do get into circulation. While ROS may play an integral role in overall adaptations to exercise, these studies suggest that the beneficial effects of ROS on muscle may be more local rather than exerting a direct effect on systemic adaptations.

Exercise sets in motion adaptive signaling partly through cellular ROS acting as second messengers to alter redox sensitive enzymes (Sadoshima, 2006). Following exercise the cellular redox status transitions toward a more oxidized environment, leading to the activation of endogenous protective mechanisms (Frasier et al., 2011a, 2013). The ROS generated during exercise likely play a major role in the upregulation of antioxidant defense mechanisms, as evidenced by the increase in GR that results after acute exercise. Importantly this up-regulation is abrogated in cardiac muscle when NOX2-generated ROS are blocked prior to exercise (Frasier et al., 2013). ROS also play an important component in the nuclear translocation of Nrf2 and its binding activity as a transcription factor in cardiac muscle. The importance of this response following 2 days of exercise is demonstrated in Nrf2 knockout mice that display an exacerbated oxidative stress response to exercise with a decrease in key antioxidant enzymes and precursors (Muthusamy et al., 2012). Similarly, in skeletal muscle ROS appear to be important for the activation of transcription factors for mitochondrial biogenesis, as daily vitamin C and E supplementation negated the increase in cytochrome c oxidase subunit IV and PGC-1α following 11 weeks of endurance training in humans (Richters et al., 2011). Further support for *in vivo* local ROS production by the exercising skeletal muscle has been provided by microdialysate samples that were analyzed for cytochrome c reduction as a marker of superoxide production (Hellsten et al., 2007). It has also been shown that skeletal muscle produced ROS may facilitate angiogenesis and increased capillary density through enhanced VEGF production (Kosmidou et al., 2001), but *in vivo* findings with antioxidant supplementation indicate that ROS may not be an important component to the VEGF response from exercise (Hellsten et al., 2007). Evidence for direct ROS-mediated adaptations is difficult pin down due to their promiscuous nature, but the aftermath of an increased oxidized environment is a tell-tale sign of ROS-mediated effects. These studies provide strong evidence for a direct effect of ROS on the enhanced antioxidant response to exercise and indicate this as one of the primary adaptive signaling events in both cardiac and skeletal muscle.

### Compensatory changes in endogenous antioxidant defenses and oxidative phosphorylation capacity

In regard to adaptations, ROS production during exercise may act as a prophylactic in the mitigation of a greater oxidative stress, as in myocardial ischemia/reperfusion injury, or skeletal muscle oxidative stress during nutrient overload. It is well documented in the exercise cardioprotection literature that acute exercise improves the redox buffering capacity of the cell as evidenced by an increase in redox specific enzymes such as glutathione reductase (GR), thioredoxin reductase, and MnSOD (Yamashita et al., 1999; Frasier et al., 2011b, 2013; Lee et al., 2012b; Fisher-Wellman et al., 2013). Changes in the activity of H_2_O_2_ scavenging systems are less robust in the response to exercise, with most studies finding little to no difference in glutathione peroxidase, catalase, and thioredoxin (Frasier et al., 2011a). An enhanced ability to cope with an oxidative stress of greater magnitude is responsible for a large portion of the exercise-induced adaptations afforded by exercise. Several studies have demonstrated that when antioxidants are administered prior to exercise, the protection against myocardial infarction and maintenance of mechanical function following I/R are lost (Yamashita et al., 1999; Akita et al., 2007; Nelson et al., 2011). This implies that ROS are acting through redox circuits during an exercise bout to modify the cellular response to that stressor; and without this signal, some adaptations are lost. However, the locus of ROS production for cardiac adaptions to exercise is still under investigation. We recently found that cytoplasmic ROS generated by NOX2 increases GR activity through post-translational modifications, and the NOX2 inhibitor apocynin abolished the increase in activity immediately after, and 24 h after exercise. While not all studies have shown ROS to be a requirement for cardioprotection (Taylor and Starnes, 2012), it is hard to refute the evidence that there are beneficial ROS that can lead to favorable adaptions.

It is well known that exercise training increases skeletal muscle mitochondrial density, with a subsequent increase in aerobic capacity. But of equal importance is the quality and oxidative phosphorylation capacity of individual mitochondrion. Rats bred for high-capacity running have increased maximal ADP-stimulated respiration and greater complex IV capacity compared to rats bred for low-capacity running (Tweedie et al., 2011). Although the high-capacity runners have higher basal rates of H_2_O_2_ emission in soleus muscle, they also exhibit a compensatory increase in SOD activity. Furthermore, they had lower DNA damage as assessed by 8-OHdG. These findings are important as a clue to skeletal muscle adaptations to exercise, as enhanced exercise capacity is linked with a favorable compensation to ROS scavenging. Enhanced respiratory capacity as an adaptation to exercise permits cellular energetics to be better maintained, with fewer limitations on the system, and findings in permeabilized fibers from sedentary humans undergoing endurance and strength training programs support this as well. Following endurance and strength training, oxidative phosphorylation capacity increases with the greatest shift in capacity arising from fatty acid oxidation (Pesta et al., 2011). Similar to skeletal muscle, enhancements in cardiac mitochondrial phosphorylation capacity and efficiency was demonstrated in a mouse model of diet-induced obesity and exercise. Isolated cardiac mitochondria from mice exposed to a high-fat diet and 8–10 weeks of high-intensity interval training displayed higher maximal respiratory capacity and ADP phosphorylation to oxygen consumption (P/O) ratios (Hafstad et al., 2013) compared to sedentary counterparts. Importantly, these adaptations resulted in significant improvements in LV function and reductions in myocardial fibrosis. We have also observed compensatory responses to the antioxidant network from exercise and high calorie diets in heart and skeletal muscle, whereby thioredoxin reductase-2 plays an important role in modulating the levels H_2_O_2_ produced during fatty acid oxidation (Fisher-Wellman et al., 2013). Insight from these studies demonstrates that mitochondrial adaptations/maladaptations are involved in the etiology of cardiometabolic abnormalities, and antioxidant defense mechanisms and respiratory capacity play an important component in affected tissues. In combination, adaptations to these components allows for tighter regulation of redox- and energy-homeostasis during episodes of increased oxidative stress that is encountered with disease pathology.

## Reconciling the paradox: the road ahead

The mechanisms underlying this contrast in outcomes between therapeutically-induced and disease-induced oxidative stress are not clear, as differences in source of ROS, sub-cellular and tissue compartmentalization, temporality of ROS production (i.e., pulsatile vs. constitutive), and transcriptional response pathways are all likely involved to some extent. Specific pathways that have been shown to be involved in mediating the adaptive responses to oxidative stress in striated muscle include activation of ROS scavenging networks, increases in mitochondrial biogenesis/respiratory capacity, and activation of cell quality control systems (e.g., autophagy/mitophagy). When oxidative stress is prolonged or of high enough magnitude in disease settings, the myocyte still responds in a controlled manner; only this response is a result of a highly oxidized environment that clearly results in a maladaptive phenotype. Overwhelming levels of oxidative stress in disease states leads to alterations in myocyte mitochondrial function and polarity, cell death, and hyper-activation of neutrophils/macrophages with subsequent fibrosis.

Unfortunately the contrast between beneficial ROS and maladaptive ROS may not be as clear cut as originally thought due to the complexity of regulation in biological redox signaling. From a therapeutic standpoint, this ambiguity makes it very difficult to implement antioxidant therapies in the clinic, as there is now very compelling evidence that antioxidants exert negative effects under certain physiological contexts, and in some cases may actually do more harm than good (Ristow, 2014). Moreover, it is now clear that a fine regulation of redox signaling is layered on top of gene regulation and extracellular-regulated responses to oxidative stress. For example, recent findings concerning the role of “mitohormesis” in maintaining homeostasis, particularly under various contexts of nutrient deprivation and overload, serve to add another layer of complexity to the role of redox signaling in the cell (Ristow and Schmeisser, 2014; Yun and Finkel, 2014). Never-the-less, these complexities only serve to underscore the importance of understanding how cellular networks respond to oxidative challenges. If we are to move forward in our comprehension of these networks then there has to be a more rigorous evaluation of the specific intra/intercellular systems that are evoked by ROS, and the key signaling events that are involved. Steps in this direction will further our understanding as to what controls the switch in response to beneficial vs. harmful oxidative stress. Uncovering these regulatory control nodes may 1 day lead to advances that allow us to remain in the Goldilocks Zone en route to dampening the burden of cardio-metabolic disease.

### Conflict of interest statement

The authors declare that the research was conducted in the absence of any commercial or financial relationships that could be construed as a potential conflict of interest.
